# Modulation of Cytokines Production by Indomethacin Acute Dose during the Evolution of Ehrlich Ascites Tumor in Mice

**DOI:** 10.1155/2015/924028

**Published:** 2015-08-12

**Authors:** Luciana Boffoni Gentile, Nicolle Queiroz-Hazarbassanov, Cristina de Oliveira Massoco, Denise Fecchio

**Affiliations:** ^1^Department of Pathology, School of Medicine, São Paulo State University (UNESP), 18618-970 Botucatu, SP, Brazil; ^2^Laboratory of Glycobiology, Carlos Chagas Filho Biophysics Institute (IBCCF), Federal University of Rio de Janeiro (UFRJ), 21941-902 Rio de Janeiro, RJ, Brazil; ^3^Applied Pharmacology and Toxicology Laboratory, School of Veterinary Medicine, University of São Paulo, 05508-900 São Paulo, SP, Brazil

## Abstract

The aim of the present study was to investigate the influence of a nonselective COX1/COX2 inhibitor (indomethacin) on tumor growth of Ehrlich Ascites Tumor (EAT) in mice, using as parameters the tumor growth and cytokine profile. Mice were inoculated with EAT cells and treated with indomethacin. After 1, 3, 6, 10, and 13 days the animals were evaluated for the secretion of TNF*α*, IL-1*α*, IL-2, IL-4, IL-6, IL-10, and IL-13 and PGE_2_ level in peritoneal cavity. The results have shown that EAT induces PGE_2_ production and increases tumor cells number from the 10th day. The cytokine profile showed EAT induces production of IL-6 from 10th day and of IL-2 on 13th day; the other studied cytokines were not affected in a significant way. The indomethacin treatment of EAT-bearing mice inhibited the tumor growth and PGE_2_ synthesis from the 10th day. In addition, the treatment of EAT-bearing mice with indomethacin has stimulated the IL-13 production and has significantly inhibited IL-6 in the 13th day of tumor growth. Taken together, the results have demonstrated that EAT growth is modulated by PGE_2_ and the inhibition of the tumor growth could be partly related to suppression of IL-6 and induction of IL-13.

## 1. Introduction

In tumor microenvironment, the relationship between the inflammatory, stromal, neoplastic cells and chemical mediators produced in the neoplasm site is complex and is the key for the disease outcome. Inflammation orchestrates the microenvironment by resident and recruited leukocytes which are responsible to drive a delicate balance between antitumor immunity and tumor-originated inflammatory activity [1]. One example of this regulation is the macrophage polarization driven by tumor microenvironment towards a suppressive phenotype or the known type 2 macrophage (M2) which is a consequence of presence of immunosuppressive factors (e.g., IL-10 and TGF-*β*) [2]. In addition, chemical mediators produced by tumor cells and/or immune cells such as IL-1, IL-4, IL-6, IL-10, IL-13, and prostaglandin (PG) E_2_ can directly suppress the cytotoxic action of macrophages, inhibiting both their migration toward the chemotactic stimuli and their adherence to endothelial cells, or indirectly by modulating the immune response by stimulating macrophages to produce PGE_2_ [3]. Moreover, in carcinomas the suppression of Th1-type response is usual, specific for tumors, due to Th2 cytokines production by T cells such as IL-4 and IL-10, facilitating neoplastic growth [4].

Several physiological and pathophysiological responses, including tumor growth and promotion, are modulated by metabolites of arachidonic acid. Arachidonic acid is the precursor for the biosynthesis of all the eicosanoid messengers, such as prostaglandins, thromboxanes, leukotrienes, lipoxins, and hydroxyeicosatetraenoic acids (HETEs). PGE_2_ is a primary product of arachidonic metabolism and is synthesized via the cyclooxygenase (COX) and prostaglandin synthase pathways. Such prostaglandin is a major eicosanoid detected in inflamed tissues, responsible for much of the proinflammatory effects and suppression of some host defense mechanisms against the neoplastic growth [5].

The use of nonsteroidal anti-inflammatory drugs (NSAIDs) which prevent PGE_2_ production has been associated with reduced risk of some types of human tumors, including breast, lung, colon, head, and neck due to inhibition of prostaglandin synthesis [6]. Indomethacin, a nonselective COX1/COX2 inhibitor and widely used NSAID, can alter phospholipid profiles of invasive human breast cancer cells towards a less invasive phospholipid profile, reducing the invasive and metastatic behavior of the neoplastic cells [7]. A variety of studies with laboratory animals and clinical research have demonstrated whether NSAIDs suppress cancer growth to increase the anticancer effects of current chemotherapy in different types of cancer. However, it was established that the inflammatory reaction in the neoplastic tissue is of lower intensity as compared to normal tissues [8].

The use of experimental models has contributed to evaluating the engineered inflammatory response against tumors, by determining the types of inflammatory cells, and tumor immunity, as well as cytokines released during the establishment of the tumor. Ehrlich Ascites Tumor (EAT) has been used as a model to evaluate the inflammatory response against the tumor growth because of its development in all mice strains and easy manipulation. During the EAT development intense influx of polymorphonuclear leukocytes occurs as well as increase of the number and cytotoxic activity of NK cells, which is inhibited by the treatment with indomethacin, suggesting the participation of prostaglandins in this process [9]. It has also been shown that during EAT growth there is a large release of PGE_2_ without activation of peritoneal macrophages, suggesting that this prostaglandin, which may be synthesized primarily by the neoplastic cells, is suppressing the inflammatory response against the tumor because of its high concentrations in the tumor site [7].

According to this context, the aim of this study was to analyze and characterize the cytokines profile and the involvement of PGE_2_ in the evolution of EAT.

## 2. Material and Methods

### 2.1. Animals

Four- to six-week-old male Swiss mice, from our animal facilities, were used. All animal procedures were in accordance with the ethical principles adopted by the Brazilian College of Animal Experimentation and approved by the Ethical Committee for Animal Research of School of Medicine, Universidade Estadual Paulista (UNESP) (Protocol number 17/99).

### 2.2. Ehrlich Ascites Tumor (EAT)

The tumor was maintained in Swiss mice in the ascitic form. Tumor cells were collected by aspiration with a Pasteur pipette, centrifuged for 10 min at 200 g, and washed twice with phosphate-buffered saline (PBS, pH 7.2). Cell viability was evaluated by trypan blue (0.2%) exclusion test and only cell suspensions with more than 95% viability were used. In all experimental protocols, mice were injected intraperitoneally with 1 × 10^3^ tumor cells per animal.

### 2.3. Drug Treatment

Indomethacin (Sigma) was dissolved in Tris buffer 1 M, pH 8.0, and the final concentration adjusted with sterile pyrogen-free physiological saline solution at 1 mg/kg.

### 2.4. Total and Neoplastic Ascites Cell Count

Mice were euthanized (ketamine chloride and xylazine HCl), the peritoneal cavity was washed with 3 mL of PBS, the material was fixed in solution of 0.5% crystal violet dissolved in 30% glacial acetic acid, and the total number of cells (leukocytes and neoplastic cells) was determined by counting in Neubauer chamber. For differential counts, aliquots of 0.3 mL of each peritoneal washed were adjusted for 2 × 10^5^ cells/mL and centrifuged at 600 rpm during 3 minutes. The cells were fixed in methanol for 5 minutes and stained with Giemsa for identification of neoplastic cells.

### 2.5. Immunoenzymatic Assay

Samples were obtained by centrifugation of the ascites fluid at 1500 rpm for 10 minutes obtaining the supernatants which were evaluated for the cytokines analysis. The cytokines profile was obtained through Enzyme-Linked Immunosorbent Assay (ELISA) using supernatant aliquots of the peritoneal washing. The following cytokines were analyzed: TNF*α*, IL-1*α*, IL-2, IL-4, IL-6, IL-10, and IL-13. The protocols, antibodies, and reagents used were according to the manufacturer's specifications (R&D System).

### 2.6. Experimental Design

**Figure 1:**
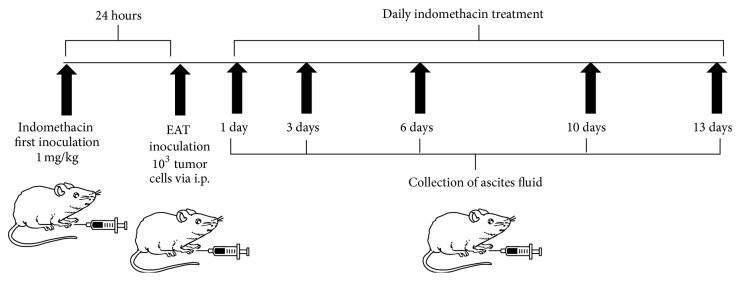
Study setting and time period. To assess the involvement of prostaglandin in cytokines profile during growth of EAT, mice were inoculated with 1000 tumor cells intraperitoneally (i.p.) and received indomethacin at a dose of 1 mg/kg, once a day, via i.p. The first dose of indomethacin was administered 24 hours prior to tumor implantation. After 1, 3, 6, 10, and 13 days the animals were euthanized and evaluated for cytokine profile.

Mice were inoculated with 1 × 10^3^ tumor cells intraperitoneally (i.p.) and received indomethacin at a dose of 1 mg/kg, once daily, by i.p. injection. The first dose of indomethacin was administered 24 hours before tumor implantation. After 1, 3, 6, 10, and 13 days the animals were euthanized, the ascites fluid was collected and centrifuged, and the cell population was evaluated for total number of neoplastic cells, present in the peritoneal cavity. The ascitic fluid supernatant was separated and stored at −20°C for later determination of cytokines. As control, EAT-bearing mice were treated with diluent solution (Tris buffer 1 M, pH 8.0) in the same condition as the experimental group (Figure 1).

The experimental groups were as follows: tumor-bearing animals inoculated with diluent (Vehicle; *N* = 103) and tumor-bearing animals treated with indomethacin (Indomethacin; *N* = 106).

### 2.7. Statistical Analysis

Data were analyzed by Kruskal Wallis test for independent samples and differences between groups were analyzed by Dunn's test and Mann-Whitney test. The level of significance was 5%.

## 3. Results

### 3.1. Effects of Indomethacin on the Number of Total and Neoplastic Cells

**Figure 2:**
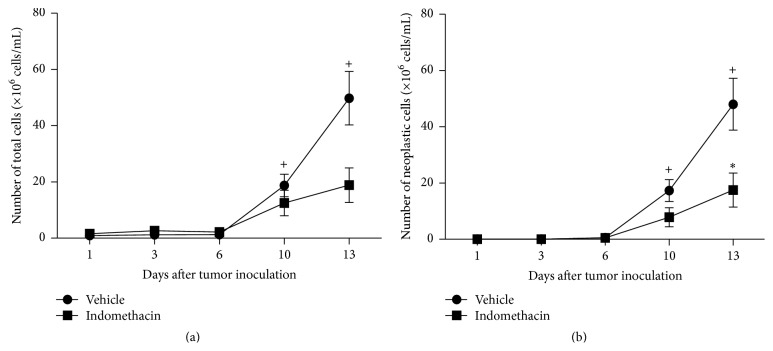
Graphs representing the number of total cells (a) and neoplastic cells (b) present in the peritoneal cavity 1, 3, 6, 10, and 13 days after inoculation of 10^3^ EAT cells in mice treated with indomethacin (Indomethacin) or diluent (Vehicle). The groups consist of 6 to 21 animals. The graphs represent the median values obtained for the variables. *P* values are provided as follows: ^+, *∗*^ *P* < 0.05, when compared to prior time point in the same experimental group or to the other experimental group in the same time point, respectively.

The total number of cells in the peritoneal cavity of EAT-bearing mice treated or not with indomethacin progressively increased from the 10th day of development. Treatment with indomethacin promoted a significant decrease in the number of tumor cells only after 13 days of implantation of neoplastic cells (Figures 2(a) and 2(b)).

### 3.2. Effects of Indomethacin on PGE_2_ and Cytokines Profile

**Figure 3:**
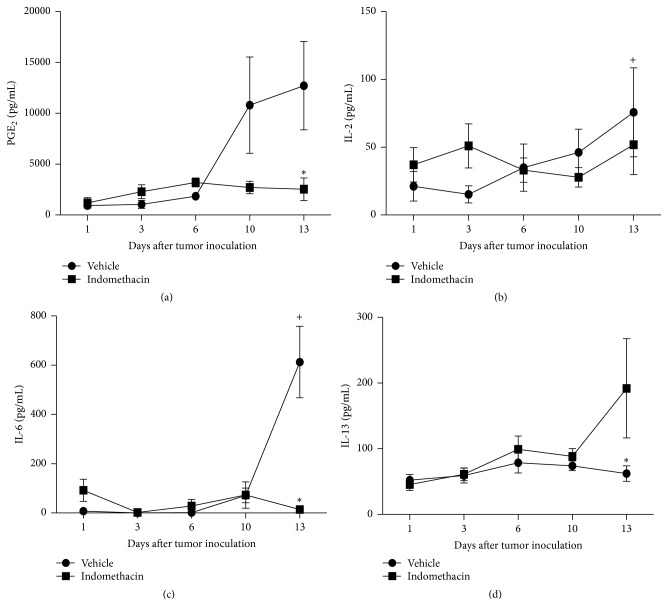
Graphs representing the levels of PGE_2_ (a), IL-2 (b), IL-6 (c), and IL-13 (d) present in the peritoneal cavity 1, 3, 6, 10, and 13 days after inoculation of 10^3^ EAT cells in mice treated with indomethacin (Indomethacin) or diluent (Vehicle). The groups consist of 6 to 21 animals. The graphs represent the median values obtained for the variables. *P* values are provided as follows: ^+, *∗*^ *P* < 0.05, when compared to prior time point in the same experimental group or to the other experimental group in the same time point, respectively.

EAT stimulated PGE_2_ production from the 10th day of tumor growth. Treatment of tumor-bearing animals with indomethacin resulted in significant inhibition of PGE_2_ only on the 13th day of neoplastic development, although, at the 10th day, the results suggest that the synthesis of the mediator was already inhibited (Figure 3(a)).

The inoculation of EAT stimulated IL-2 release on the 13th day of neoplasm evolution and the treatment with indomethacin did not affect the levels of this cytokine in tumor-bearing animals (Figure 3(b)). EAT growth stimulated release of IL-6 in the 13th day of tumor growth, whereas treatment with indomethacin of tumor-bearing animals inhibited the release of this cytokine at the same time of tumor growth (Figure 3(c)).

The neoplastic growth has not affected the IL-13 production throughout the whole experiment. EAT-bearing animals treated with indomethacin showed a significant increase in IL-13 only in the 13th day of neoplasm growth (Figure 3(d)).

**Figure 4:**
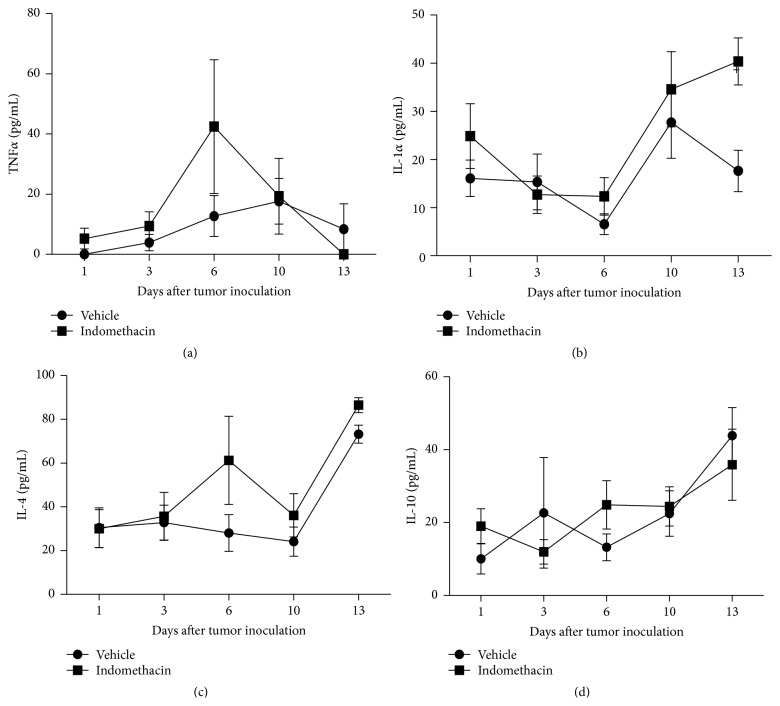
Graphs representing the levels of TNF*α* (a), IL-1*α* (b), IL-4 (c), and IL-10 (d) present in the peritoneal cavity 1, 3, 6, 10, and 13 days after inoculation of 10^3^ EAT cells in mice treated with indomethacin (Indomethacin) or diluent (Vehicle). The groups consist of 6 to 21 animals. The graphs represent the median values obtained for the variables.

There were no significant differences in the TNF*α* profile in any of the groups and times studied (Figure 4(a)). The results demonstrated that EAT did not stimulate the release of IL-1*α* during its development (Figure 4(b)). The development of EAT in animals did not stimulate the production of IL-4 throughout the experiment (Figure 4(c)). In tumor-bearing animals tumor growth has not stimulated the release of IL-10, and this cytokine profile was not modified by the treatment with indomethacin (Figure 4(d)).

## 4. Discussion

The aim of this work was to study the modulation of cytokines production by PGE_2_ suppression during the development of EAT. The analysis of nucleated cells in the peritoneal cavity revealed that tumor-bearing animals showed a progressive increase in this number from the 10th day of tumor development. The treatment of EAT-bearing mice with indomethacin induced a decrease in the total number of peritoneal cells from the same period. In order to confirm the EAT growth inhibition by indomethacin the number of tumor cells was determined. The results showed that the treatment with indomethacin of EAT-bearing animals inhibited the tumor growth in 93.3% and 65.8% in 10th and 13th days of neoplastic development, respectively. Although on 10th day of tumor growth the decrease of tumor cells in the group of mice treated with indomethacin was not statistically significant, in this group of 20 animals, 15 showed intense inhibition of neoplasm growth. These results are consistent with data from the literature, where most of the growth of transplantable mammary tumors in rodents is inhibited by indomethacin [10, 11].

The analysis of PGE_2_ levels in the peritoneal cavity of EAT-bearing animals revealed an increase of the concentration of this eicosanoid with the progressive tumor growth. Results obtained in our laboratory (unpublished data) showed that EAT cells cultured* in vitro* produce PGE_2_. It is noteworthy that the treatment of tumor-bearing animals with indomethacin reduced the concentration of PGE_2_ from the 10th day of treatment. These results suggest that PGE_2_ is associated with tumor proliferation, growth, and expansion, confirming the involvement of prostaglandins in control of tumor growth* in vivo*, both in humans and in experimental animals [12, 13].

The results obtained in the measurement of cytokines showed that the EAT promoted release of IL-2 and IL-6 in the 13th day of tumor development. However, levels of IL-1*α*, IL-4, IL-10, IL-13, and TNF*α* were not significantly altered by tumor growth.

There are numerous studies demonstrating the role of IL-1*α* as a stimulatory factor of cellular proliferation, growth, and differentiation [14–17] and its role in the invasion and metastasis of various solid tumors [18–20]. However, there were no significant differences in the profile of this cytokine in any of the time points evaluated during the EAT growth.

Although TNF*α* induces hemorrhagic necrosis in certain solid tumors, intraperitoneal tumors, such as Ehrlich tumor, are resistant to its cytotoxic effect [21]. There was no production of this cytokine in animals with EAT in any time during tumor development.

After 13 days of EAT growth there was a significant increase in the concentration of IL-2. One aspect of the importance of IL-2 is its use in immunotherapy for cancer; there are several studies demonstrating its effectiveness in treating human tumors such as renal cancer, metastatic melanoma, and murine tumors such as Ehrlich Ascites Tumor and B16F10 melanoma [22–25]. In the specific case of EAT 90% of tumor-bearing mice were cured with a combination therapy consisting of the administration of indomethacin and IL-2 [24]. Despite the increase in the IL-2 levels after 13 days of tumor development, the treatment of tumor-bearing mice with indomethacin has not altered the production of this cytokine during the neoplastic growth. This result indicates the suppression of PGE_2_ has not modulated the IL-2 levels and the decrease in the number of tumor cells from the 10th day observed with the indomethacin treatment should not be associated with IL-2.

Although there are several studies showing that IL-4 can inhibit tumor growth, induce apoptosis in neoplastic cells, stimulate the activity of antigen-presenting cells in patients with cancer, and regulate the expression of adhesion molecules in neoplastic cells [26], the levels of IL-4 were not changed during the development of EAT. Although not significant, a tendency to increase in the concentration of IL-4 was verified on the 13th day of development of the EAT, which could be reflecting a Th2 immune response against the tumor [27].

Increased levels of IL-6 observed from the 10th day of development of the EAT corroborate the results obtained by da Silva et al. [28], who found a significant increase in IL-6 at similar times. Chen et al. [29] detected in squamous cell carcinoma of head and neck* in situ* IL-6, confirming the role of this cytokine in immune unresponsiveness and carcinogenesis. Nakano et al. [30] also detected elevated levels of IL-6 in tissues of homogeneous oral squamous cell carcinoma. Based on these findings, IL-6 could have a role in host immune suppression and induction of cachexia in times of late neoplastic growth in animals with EAT. The microenvironments of solid and ascites tumors are crucial players for the tumor growth. Perhaps this cytokine is being produced by either neoplastic or late-phase response inflammatory cells as in animals with EAT the release of IL-6 from the 10th day of tumor growth is increased. Fibroblast-like stromal cells modulate cancer cells through secreted factors and adhesion, but those factors are not fully understood. Using a cell coculture system, a group of researchers have identified critical stromal factors that modulate cancer growth positively and negatively, showing that gastric stromal cells secreted IL-6 as a growth and survival factor for gastric cancer cells [31].

In addition, PGE_2_ and IL-6 signal pathways are involved in the cross talk between tumor and stromal cells [32]. Increased PGE_2_ levels induce the expression of the inflammatory factor IL-6 in fibroblasts [33, 34]. IL-6 is an important mediator involved in the cross talk between cancer-associated fibroblasts (CAFs) and tumor cells [35, 36]. Apparently, PGE_2_ induces the increase of tumor cells number, and this effect is dependent on cytokines and IL-6 seemed to be the most important in EAT development. The results in EAT-bearing mice have shown increase in the PGE_2_ levels from the 10th day and IL-6 levels on 13th day. These results may suggest that IL-6 was induced by PGE_2_. Although the presence of CAFs in ascites tumors is not an obvious finding, in the current experimental model, IL-6 could be responsible for the cross talk between CAFs or phenotypically similar cells and tumor cells [32]. Then, when the levels of PGE_2_ and IL-6 decreased by the use of indomethacin this cross talk is disrupted and the sequential EAT growth inhibition has occurred. Besides, results obtained in the IL-6 levels in EAT-bearing animals treated with indomethacin allow us to suggest that the decrease in the release of this cytokine could be related to inhibition of tumor growth, as in tumor-bearing animals there was no increase in IL-6 from the 10th day of tumor growth, when there is tumor growth, with increased numbers of tumor cells and increased production of PGE_2_.

IL-10 is a potent immunosuppressive cytokine that has been found to play a role in a variety of human neoplasms, including gastric, colorectal, and prostate cancers [37–40]. Despite the findings in these neoplastic types, the results have demonstrated no stimulation for the release of IL-10 in EAT-bearing animals.

Reports in the literature associated IL-13 with inhibition of tumor proliferation [41], by increasing antigen presentation to T cells [42]. In addition, IL-13, among other functions, stimulates proliferation of B cells and cytotoxic T cells. Treatment of animals with EAT with indomethacin promoted a significant increase in the level of IL-13 on the 13th day of neoplastic changes, coinciding with the inhibition of tumor growth. The increase in IL-13 observed in EAT carrier animals treated with indomethacin could be due to the change of Th2 CD4 T cells and/or increase in antigen presentation by macrophages. However, further studies are necessary for clarifying this issue.

## 5. Conclusions

The results obtained allow us to infer that the EAT inoculation stimulated PGE_2_ production throughout its development and that the neoplasm growth has a positive influence on the production of IL-2 and IL-6 in the 13th day of tumor development. In addition, the treatment with indomethacin of EAT-bearing animals positively modulates the production of IL-13, negatively the release of IL-6, and does not alter the profiles of IL-1*α*, IL-2, IL-4, IL-10, and TNF*α*. Also, the indomethacin significantly inhibited the synthesis of PGE_2_ on the 13th day of neoplastic development.

In conclusion, our findings showed that PGE_2_ modulates the growth of EAT and that inhibition of tumor growth could be partly related to suppression of the release of IL-6 and to stimulation of the release of IL-13. Additional studies* in vivo* and* in vitro* using specific inhibitors for COX-2, assessing the relationships between microenvironment cell subpopulations, cytokines, and neoplastic cells in tumor growth inhibition, may contribute to the elucidation of the mechanism(s) by which PGE_2_ modulates the development of the EAT.

## References

[1] Ben-Baruch A. (2006). Inflammation-associated immune suppression in cancer: the roles played by cytokines, chemokines and additional mediators. *Seminars in Cancer Biology*.

[2] Allavena P., Sica A., Garlanda C., Mantovani A. (2008). The Yin-Yang of tumor-associated macrophages in neoplastic progression and immune surveillance. *Immunological Reviews*.

[3] Zeidler R., Csanady M., Gires O., Lang S., Schmitt B., Wollenberg B. (2000). Tumor cell-derived prostaglandin E2 inhibits monocyte function by interfering with CCR5 and Mac-1. *The FASEB Journal*.

[4] Yamamura M., Modlin R. L., Ohmen J. D., Moy R. L. (1993). Local expression of antiinflammatory cytokines in cancer. *The Journal of Clinical Investigation*.

[5] Raz A., Levine G., Khomiak Y. (2000). Acute local inflammation potentiates tumor growth in mice. *Cancer Letters*.

[6] Patrignani P. (2000). Nonsteroidal anti-inflammatory drugs, COX-2 and colorectal cancer. *Toxicology Letters*.

[7] Natarajan K., Mori N., Artemov D., Aboagye E. O., Chacko V. P., Bhujwalla Z. M. (2000). Phospholipid profiles of invasive human breast cancer cells are altered towards a less invasive phospholipid profile by the anti-inflammatory agent indomethacin. *Advances in Enzyme Regulation*.

[8] Fecchio D., Sirois P., Russo M., Jancar S. (1990). Studies on inflammatory response induced by Ehrlich tumor in mice peritoneal cavity. *Inflammation*.

[9] Lala P. K., Santer V., Libenson H., Parhar R. S. (1985). Changes in the host natural killer cell population in mice during tumor development. 1. Kinetics and ‘in vivo’ significance. *Cellular Immunology*.

[10] Diament M. J., Peluffo G. D., Stillitani I. (2006). Inhibition of tumor progression and paraneoplastic syndrome development in a murine lung adenocarcinoma by medroxyprogesterone acetate and indomethacin. *Cancer Investigation*.

[11] Blidner A. G., Salatino M., Mascanfroni I. D. (2015). Differential response of myeloid-derived suppressor cells to the nonsteroidal anti-inflammatory agent indomethacin in tumor-associated and tumor-free microenvironments. *Journal of Immunology*.

[12] Qian X., Gu L., Ning H. (2013). Increased Th17 cells in the tumor microenvironment is mediated by IL-23 via tumor-secreted prostaglandin E_2_. *The Journal of Immunology*.

[13] Mao Y., Sarhan D., Steven A., Seliger B., Kiessling R., Lundqvist A. (2014). Inhibition of tumor-derived prostaglandin-E2 blocks the induction of myeloid-derived suppressor cells and recovers natural killer cell activity. *Clinical Cancer Research*.

[14] Huber M., Rutherford A., Meister W., Weiss A., Röllinghoff M., Lohoff M. (1996). TCR- and IL-1-mediated co-stimulation reveals an IL-4-independent way of Th2 cell proliferation. *International Immunology*.

[15] Voronov E., Reich E., Dotan S. (2010). Effects of IL-1 molecules on growth patterns of 3-MCA-induced cell lines: an interplay between immunogenicity and invasive potential. *Journal of Immunotoxicology*.

[16] Cheng J., Li L., Liu Y., Wang Z., Zhu X., Bai X. (2012). Interleukin-1*α* induces immunosuppression by mesenchymal stem cells promoting the growth of prostate cancer cells. *Molecular Medicine Reports*.

[17] van Nieuwenhoven F. A., Hemmings K. E., Porter K. E., Turner N. A. (2013). Combined effects of interleukin-1*α* and transforming growth factor-*β*1 on modulation of human cardiac fibroblast function. *Matrix Biology*.

[18] Furuya Y., Ichikura T., Mochizuki H. (1999). Interleukin-1alpha concentration in tumors as a risk factor for liver metastasis in gastric cancer. *Surgery Today*.

[19] Tomimatsu S., Ichikura T., Mochizuki H. (2001). Significant correlation between expression of interleukin-1*α* and liver metastasis in gastric carcinoma. *Cancer*.

[20] Carmi Y., Rinott G., Dotan S. (2011). Microenvironment-derived IL-1 and IL-17 interact in the control of lung metastasis. *The Journal of Immunology*.

[21] Manda T., Shimomura K., Mukumoto S. (1987). Recombinant human tumor necrosis factor-*α*: evidence of an indirect mode of antitumor activity. *Cancer Research*.

[22] Margolin K. A. (2000). Interleukin-2 in the treatment of renal cancer. *Seminars in Oncology*.

[23] Saraceni C., Agostino N., Weiss M. J., Harris K., Nair S. (2015). Clinical characteristics and treatment-related biomarkers associated with response to high-dose interleukin-2 in metastatic melanoma and renal cell carcinoma: retrospective analysis of an academic community hospital’s experience. *SpringerPlus*.

[24] Lala P. K., Parhar R. S., Singh P., Lala P. K. (1990). Cure of murine Ehrlich ascites tumors with chronic oral indomethacin therapy combined with intraperitoneal administration of LAK cells and IL-2. *Cancer Letters*.

[25] de Galdeano A. G., Cruz-Conde J. C., Boyano M. D., García-Vázquez M. D., Cañavate M. L. (2001). Effect of IL-2 and IL-6 on parameters related to metastatic activity in a murine melanoma. *Pathobiology*.

[26] Gocheva V., Wang H.-W., Gadea B. B. (2010). IL-4 induces cathepsin protease activity in tumor-associated macrophages to promote cancer growth and invasion. *Genes & Development*.

[27] Segura J. A., Barbero L. G., Márquez J. (1997). Early tumor effect on splenic Th lymphocytes in mice. *FEBS Letters*.

[28] da Silva R. J., da Silva M. G., Vilela L. C., Fecchio D. (2002). Antitumor effect of *Bothrops jararaca* venom. *Mediators of Inflammation*.

[29] Chen Z., Malhotra P. S., Thomas G. R. (1999). Expression of proinflammatory and proangiogenic cytokines in patients with head and neck cancer. *Clinical Cancer Research*.

[30] Nakano Y., Kobayashi W., Sugai S., Kimura H., Yagihashi S. (1999). Expression of tumor necrosis factor-*α* and interleukin-6 in oral squamous cell carcinoma. *Japanese Journal of Cancer Research*.

[31] Kawada M., Inoue H., Ohba S. (2015). Stromal cells positively and negatively modulate the growth of cancer cells:stimulation via the PGE_2_-TNF*α*-IL-6 pathway and inhibition via secreted GAPDH-E-cadherin interaction. *PLOS ONE*.

[32] Li P., Shan J. X., Chen X. H. (2015). Epigenetic silencing of microRNA-149 in cancer-associated fibroblasts mediates prostaglandin E2/interleukin-6 signaling in the tumor microenvironment. *Cell Research*.

[33] Zhang Y., Lin J.-X., Vilcek J. (1988). Synthesis of interleukin 6 (interferon-*β*2/B cell stimulatory factor 2) in human fibroblasts is triggered by an increase in intracellular cyclic AMP. *The Journal of Biological Chemistry*.

[34] Hinson R. M., Williams J. A., Shacter E. (1996). Elevated interleukin 6 is induced by prostaglandin E2 in a murine model of inflammation: possible role of cyclooxygenase-2. *Proceedings of the National Academy of Sciences of the United States of America*.

[35] Erez N., Truitt M., Olson P., Arron S. T., Hanahan D. (2010). Cancer-associated fibroblasts are activated in incipient neoplasia to orchestrate tumor-promoting inflammation in an NF-*κ*B-dependent manner. *Cancer Cell*.

[36] Quante M., Tu S. P., Tomita H. (2011). Bone marrow-derived myofibroblasts contribute to the mesenchymal stem cell niche and promote tumor growth. *Cancer Cell*.

[37] Halak B. K., Maguire H. C., Lattime E. C. (1999). Tumor-induced interleukin-10 inhibits type 1 immune responses directed at a tumor antigen as well as a non-tumor antigen present at the tumor site. *Cancer Research*.

[38] Shen Z., Seppänen H., Vainionpää S. (2012). IL10, IL11, IL18 are differently expressed in CD14^+^ TAMs and play different role in regulating the invasion of gastric cancer cells under hypoxia. *Cytokine*.

[39] Miteva L. D., Stanilov N. S., Deliysky T. S., Stanilova S. A. (2014). Significance of −1082A/G polymorphism of IL10 gene for progression of colorectal cancer and IL-10 expression. *Tumor Biology*.

[40] Wang M.-H., Helzlsouer K. J., Smith M. W. (2009). Association of IL10 and other immune response- and obesity-related genes with prostate cancer in CLUE II. *Prostate*.

[41] Kornmann M., Kleeff J., Debinski W., Korc M. (1999). Pancreatic cancer cells express interleukin-13 and –4 receptors, and their growth is inhibited by Pseudomonas exotoxin coupled to interleukin-13 and –4. *Anticancer Research*.

[42] Lopez M., Amorim L., Gane P. (1997). IL-13 induces CD34^+^ cells isolated from G-CSF mobilized blood to differentiate in vitro into potent antigen presenting cells. *Journal of Immunological Methods*.

